# Silicate-Based Electro-Conductive Inks for Printing Soft Electronics and Tissue Engineering

**DOI:** 10.3390/gels7040240

**Published:** 2021-11-27

**Authors:** Sadaf Samimi Gharaie, Amir Seyfoori, Bardia Khun Jush, Xiong Zhou, Erik Pagan, Brent Godau, Mohsen Akbari

**Affiliations:** 1 Laboratory for Innovations in Microengineering (LiME), University of Victoria, Victoria, BC V8P 5C2, Canada; s.f.sadaf@gmail.com (S.S.G.); am.seyfoori@gmail.com (A.S.); bkhunjus@uvic.ca (B.K.J.); sina81@gmail.com (X.Z.); erikpm@uvic.ca (E.P.); brentgodau@gmail.com (B.G.); 2 Center for Advanced Materials and Related Technologies (CAMTEC), University of Victoria, Victoria, BC V8P 5C2, Canada; 3 Biotechnology Center, Silesian University of Technology, 2A, 44-100 Gliwice, Poland; 4 School of Biomedical Engineering, University of British Columbia, Vancouver, BC V6T 1Z4, Canada

**Keywords:** 3D printing, electrically conductive bio-ink, Laponite, graphene oxide

## Abstract

Hydrogel-based bio-inks have been extensively used for developing three-dimensional (3D) printed biomaterials for biomedical applications. However, poor mechanical performance and the inability to conduct electricity limit their application as wearable sensors. In this work, we formulate a novel, 3D printable electro-conductive hydrogel consisting of silicate nanosheets (Laponite), graphene oxide, and alginate. The result generated a stretchable, soft, but durable electro-conductive material suitable for utilization as a novel electro-conductive bio-ink for the extrusion printing of different biomedical platforms, including flexible electronics, tissue engineering, and drug delivery. A series of tensile tests were performed on the material, indicating excellent stability under significant stretching and bending without any conductive or mechanical failures. Rheological characterization revealed that the addition of Laponite enhanced the hydrogel’s mechanical properties, including stiffness, shear-thinning, and stretchability. We also illustrate the reproducibility and flexibility of our fabrication process by extrusion printing various patterns with different fiber diameters. Developing an electro-conductive bio-ink with favorable mechanical and electrical properties offers a new platform for advanced tissue engineering.

## 1. Introduction

Engineered materials that can imitate native tissue properties are promising tools for treating a wide variety of clinical problems in patients and for use in research labs. In particular, hydrogels engineered to be conductive can be used to produce flexible, biocompatible sensors for the in vivo recording of bio-signals and biomolecules, or in tissue engineering applications [1]. However, designing hydrogels capable of generating the desired three-dimensional (3D) structures with the necessary physical properties, while being biocompatible and scalable, is nontrivial. 3D printing technology is ideal for the intensive manufacturing of these structures for in vivo and in vitro applications [2,3]. Critically, 3D printing provides high reproducibility and precise control in an automated manner, making it suitable for introducing a consistent method of production for fabrication of complex structures [4]. A limiting factor preventing the commercialization of bioprinted tissues is the capability of bio-inks in providing the requisite physical and mechanical properties during and after the printing process. A characteristic that can be useful in a variety of biomedical applications is the electroconductivity of the printed structures. Electroconductive structures are essential for the bioengineering of electro-activated tissues such as cardiac and muscle tissues. Moreover, electroconductive microstructures can be used as on-demand drug delivery platforms with targeted release of drugs in response to the external or environmental electrical stimuli [5,6]. Therefore, electrically conductive biomaterials provide a new level of control over engineering scaffolds for the stimulation of cells and producing biosensors for drug delivery applications [7]. In this way, 3D printing is a promising fabrication method for the assembly of electroconductive hydrogel layers in 3D constructs with applications in tissue engineering [8]. Bio-engineering electro-responsive tissues using 3D printing requires a bio-ink that has similar physical characteristics to the underlying tissue while being electro-conductive [9,10]. For instance, conductive hydrogels in tissue engineering applications provide uniform electrical stimuli to living cells and improve electrical signal propagation, cell adhesion, and differentiation within the desired 3D scaffold that mimics the native tissue [11]. As another example, electro-conductive hydrogel enable the production of soft electronics with a wide range of biomedical applications, including wearable sensors, smart wound dressings, and implantable diagnostic devices.

Hydrogel-based bio-inks should be flexible, physically robust, and capable of withstanding the host tissue’s physical activity and movement. Additionally, the hydrogel must have suitable electro-conductive properties for conducting currents generated by in vivo sensors or by the living tissue itself (e.g., cardiac or neural action potential). Hydrogels with shear-thinning properties are good candidates for use as inks with applications in 3D printing of tissue and soft electronics [12]. This is due to their non-Newtonian [13] viscoelastic behavior under shear stress, which makes them extrudable through existing printheads, but fairly robust in the relaxed state after extrusion. Furthermore, hydrogels can be used to print a variety of complex patterns of different sizes and are often biocompatible, making them ideal candidates for tissue engineering applications [14]. Some of the most common polymers used for printing soft structures include hyaluronic acid (HA), [15] agarose, [16] alginate, [17] gelatin methacrylate (GelMA), [18] silk proteins, [19] elastin, [20], and poly (ethylene oxide/glycol) [21]. All the aforementioned polymers have shear-thinning properties, and thus can flow more easily through narrow printing nozzles. However, their low viscosity and slow crosslinking rates reduce the shape fidelity after the fibers are extruded from a printhead [11]. Alginate is one of the most commonly used hydrogels for 3D printing. This ubiquity is due to its excellent biocompatibilityand mild ionic gelation process. [22] Due to these properties, alginate has been extensively used as a base material for many bio-ink formulations. However, alginate lacks biologically active moieties, has minimal cellular adhesion properties, and does not promote cell proliferation and differentiation [23]. Moreover, it is worth noting that alginate-based hydrogel bio-inks are oftentimes constrained by their mechanical weakness and limited shape fidelity after printing [24]. To alleviate the physical strength limitations of alginate hydrogels, silicate nanoclays have been added to alginate hydrogels [25]. Synthetic silicates are charged disks with high aspect ratios, approximately 20–30 nm in diameter and 1 nm in thickness [26]. Laponite is an example of a silicate nanoclay, and is a synthetic, lithium–magnesium–sodium silicate of the smectite group. Laponite has been added to alginate-based hydrogels to simultaneously improve the physical properties of the hydrogels and to provide a base for cellular attachment [20]. Based on the literature, the addition of Laponite to natural polymers can improve the stability of the composite and enhance the elasticity of the final pattern [27]. This improvement is hypothesized to be a result of the hydrostatic interactions between the anionic polymer (alginate) and the positive charge on Laponite’s surface that helps in stabilizing the hydrogel [28,29]. Non-covalent and reversible interactions between the components of a biomaterial can result in modular networks with distinct biological and physical properties, including viscoelastic behavior. Laponite nanoparticles and the alginate polymer interact in a reversible and non-covalent manner. This reversible interaction explains the shear-thinning property of the biomaterial. An example of alginate Laponite hydrogels for bio-printing is provided by Ahfeld et al. [30]. They blended alginate and methylcellulose with Laponite to fabricate a bio-ink and produce scaffolds utilizing the extrusion-based method of 3D plotting [30]. Similarly, Ahasan Habib et al. developed a bio-ink composed of alginate, carboxymethyl cellulose, and montmorillonite clay instead of Laponite that had suitable physical properties for printing [31]. While the silicates-enhanced alginate hydrogel bio-inks have been extensively studied for their physical properties, their electro-conductive properties, and the possibility of modification with electro-conductive material, remain unexamined [32].

Some electro-conductive materials that have been used for engineering bio-inks include metals such as gold [33], copper [33], silver [34], and organic materials such as carbon nanotubes (CNT) [8] and graphene [35,36]. For example, Zhu et al. developed a gold nanorod (GNR) gelatin methacryloyl (GelMA)-based bio-ink for printing 3D functional cardiac tissue constructs [33]. In another study, He et al. fabricated ultrathin and electrically parallel arrays of reduced graphene oxide (rGO) films on various substrates, including flexible polyethylene terephthalate (PET) films. They used these nanocomposite films as chemical and biological sensors to detect the cellular secretion of biomolecules [37]. Graphene and graphene oxide is of particular interest due to their electro-conductive properties. This is due to their ${\mathrm{SP}}^{2}$ hybridized orbital structure providing free electrons, which gives them outstanding thermal and electrical conductive properties [38]. Graphene oxide is an oxidized form of graphene with carboxylic acid and hydroxyl functional groups [39].

The use of hydrogel-based electroconductive bio-inks has the advantage of offering a soft substrate at the interface between the electronic circuit and the tissue, a feature that overcomes the issues associated with a mechanical mismatch between current flexible electronic systems and soft tissue. An ideal bio-ink needs to be printed on a substrate that is flexible, durable, and is not cytotoxic. However, providing a stretchable, durable, and cost-effective method remains a constant challenge [1]. Various substrates have been introduced to provide a flexible and biodegradable layer for producing conductive patterns. Based on the literature silk, poly(lactic-co-glycolic) acid (PLGA), poly(imide), poly(4-vinyl pyridine), and poly(styrene-block-butadiene-block styrene) have been regularly utilized to provide a flexible substrate [40]. Tunnel injection and metallic microstrips have been employed to form conformal contacts and sufficient attachment to curved surfaces. However, maintaining the attachment without affecting the physical properties of the printed pattern remains challenging. A variety of techniques, such as inkjet printing, screen printing, and contact printing [8] have been employed for printing cost-effective conductors on paper. Unfortunately, paper-based conductors are not stretchable and lose their physical strength in aqueous environments. Poly (dimethylsiloxane) (PDMS) and electrospinning techniques have also been used to produce elastic and cost-effective substrates for fabricating flexible conductors [41,42]. Although PDMS is a cost-effective material, and provides a decent degree of flexibility, the hydrophobic nature of this polymer prevents biodegradability and permeability, which are necessary traits for drug delivery and biomedical applications. Despite this, electrospinning allows for the fabrication of nanoscale fibers from a wide variety of polymer solutions, but a complex fabrication process and poor reproducibility remain major challenges in electrospinning.

In the present work, we report a novel silicate-based conductive bio-ink composed of alginate, Laponite, and rGo. This material composition provides a printable and flexible hydrogel with low electrical resistance that can be embedded into a multilayered hydrogel bed using a multistep process. The properties of the novel bio-ink, including its structural morphology, rheological characteristics, and conductivity under different physical conditions, are analyzed. Employing rGo in the structure of the bio-ink resulted in the enhanced electro-conductivity of the final hydrogel. The shear-thinning properties, electro-conductivity, and biocompatibility of this hydrogel can be used for engineering a three-dimensional network that can mimic the extracellular matrix (ECM) structures by 3D printing a desired form [43]. The other potential application of these hydrogels is in the controlled release of therapeutic agents in response to an electrical cue to provide a novel approach for instant pain relief. Furthermore, we stretched and sutured the printed bio-ink system to show their potential function in terms of providing desirable mechanical properties for further clinical applications in stretchable electronics and tissue engineering. To the best of our knowledge, we were able for the first time to produce a material composition that combines the printable rheological properties of alginate–silicate hydrogels and the electro- conductive properties of graphene oxides. The printed conductive bio-ink on a multilayered hydrogel bed generated a flexible electric conductor with potential functions in in vivo clinical applications.

## 2. Results and Discussion

### 2.1. Hydrogel Preparation

Partially exfoliated graphite crystals were used in this research. The exfoliation process involves expanding the graphite platelets up to hundreds of times in order to eliminate aggregation and improve the lubricity and flexibility of this material [11]. Partially exfoliated graphite crystals are not stable in aqueous solutions. This is due to the low energy barrier of the van der Waals forces in the graphite crystals, which translates to the sedimentation and aggregation of graphene in water [44]. In order to prevent sedimentation and produce sufficiently large energy barriers between the graphene layers, covalent or noncovalent bonds between the layers are necessary [45]. The surface modification of graphene is the most popular approach for adding the aforementioned bonds and exfoliating graphene in water [46]. Chemical oxidation is commonly used to form the necessary covalent bonds to produce graphene oxide, which is readily dispersible in water in strongly alkaline conditions [47,48]. However, the carboxylic groups of the graphene oxide produce an acidic pH, which results in restacking and the precipitation of the graphene interlayers. In order to overcome the van der Waals forces of graphene oxide, electrostatic repulsion or steric hindrance is necessary. The addition of Laponite to graphene oxide results in electrostatic repulsion, which translates to stable aqueous graphene oxide [49]. Laponite is a platelet that is 25 nm in diameter and 1 nm in height and has positive charges on its edges and negative charges on its surface [50]. The negative charge density of the surface is due to the isomorphic substitution of ${\mathrm{Mg}}^{2+}$ by ${\mathrm{Li}}^{+}$ in the crystal lattice and which imparts charge repulsion. Accordingly, Laponite shows a remarkable swelling ratio in water [51,52]. This unique replacement reaction and the charge distribution give Laponite the ability to interact electrostatically with both anionic and cationic polymers, and to modify their rheological properties. Laponite adopts a stable colloid form in water and provides an alkaline pH which improves the stable dispersion of graphene oxide [53].

**Figure 1:**
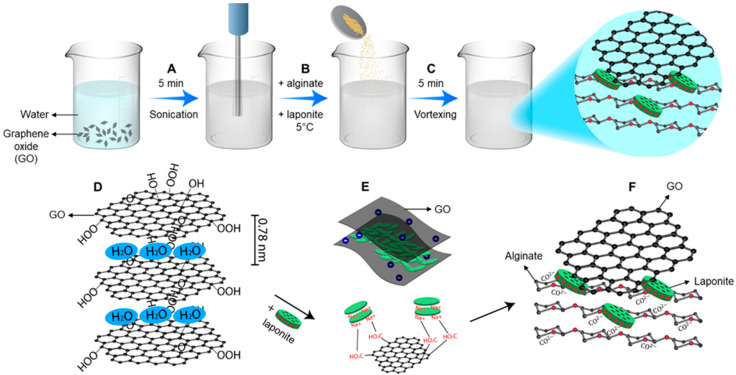
Schematic of the fabrication process of the alginate/Laponite/graphene oxide bio-ink: (**A**) preparation of a uniform colloid from 3 and 5 mg/mL graphene oxide in deionized water; (**B**) addition of alginate at room temperature and Laponite at 4 °C; (**C**) formation of a viscous nanocomposite hydrogel by vortexing; (**D**) macromolecular structure of graphene oxide dispersions in water; (**E**) electrostatic interactions of graphene oxide and Laponite; (**F**) molecular interactions of alginate/Laponite/graphene oxide.

In this study, the aqueous suspension of graphene oxide was prepared by, first, dispersing 5 and 3 mg/mL graphene oxide in deionized water and then sonicating the dispersion for 5 min to form a uniform colloid, as shown in Figure 1A. The individual concentration for each material was chosen based on the desired mechanical properties to achieve a high shape fidelity of the printed pattern, the desired cell adhesion, the electrical conductivity, and the flexibility based on the literature [28,54,55,56]. Additionally, the final concentrations were chosen based on the desired chemical, rheological, and physical properties of the hydrogel for developing shear-thinning electro-conductive bio-ink. As observed in the schematic of the sample preparation, Figure 1B, after the very gradual addition of 0.5% (*w*/*v*) alginate and vortexing the solution to dissolve all of the particles, 6% (*w*/*v*) Laponite was added to the 4 °C solutions of alginate/graphene oxide and vortexed vigorously for 5 min to form a final gel, as shown in Figure 1C. The molecular interactions of the graphene oxide in deionized water are also shown in Figure 1D. The addition of Laponite prevents the aggregation of graphene oxide in water and improves the stability of the colloid. Due to the charge repulsion between the Laponite platelets, the Laponite particles on the graphene plates prevent the platelets from aggregating, as shown in Figure 1E. Additionally, the electrostatic interactions between the negatively charged alginate and the positive charges on the Laponite nanoplatelets result in the crosslinking of the hydrogel nanocomposite and improve the mechanical properties of the hydrogel bio-ink. Fabricated nanocomposite hydrogels were also assessed in terms of cytotoxic behavior according to the ISO 10993-5 standard guideline. Cytotoxicity results were reported periodically from day 1 to day 7 and, as was displayed in Figure S1, except for the 5 mg/mL GO-containing sample extract at day 1, all the hydrogels show the highest cell viability without significant difference with the control sample (without sample extractions). The dose-dependent cytotoxic effect of the 5 mg/mL GO sample extract might be attributed to the extensive release of the GO nanosheets in the early release study (day 1), which is compensated by the medium exchange with the fresh one in subsequent days and removing the released ions from the extraction sample.

### 2.2. Chemical and Physical Characterization

#### 2.2.1. Zeta Potential

**Figure 2:**
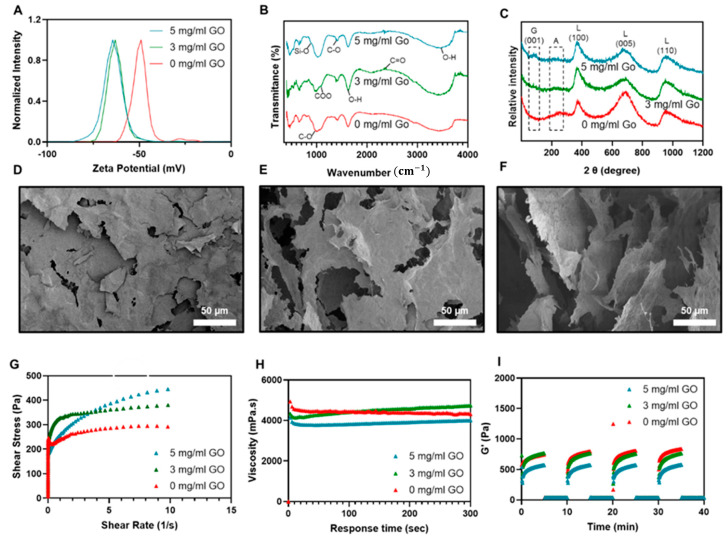
Chemical, structural, and rheological characterization of alginate/Laponite with various concentrations of graphene oxide (Go): 0, 3, and 5 mg/mL. (**A**) Zeta potential: the inset plot depicts the zeta potential of alginate/Laponite as a function of the graphene oxide concentrations. (**B**) FTIR spectra of alginate/Laponite hydrogels with various concentrations of graphene oxide. (**C**) XRD spectrum of alginate/Laponite with different concentrations of graphene oxide. (**D–F**) SEM images of alginate/Laponite bio-ink with various concentrations of graphene oxide: 0, 3, and 5 mg/mL, respectively. The overall porosity of the gel increased with increasing the concentration of graphene oxide. (**G**) Shear stress as a function of shear rate was plotted to indicate the negligible effect of adding 3 and 5 mg/mL graphene oxide. (**H**) Plot of the viscosity versus response time under constant shear rate showed a minor decrease by cooperating 5 mg/mL graphene oxide. (**I**) Elastic recovery of the bio-inks observed by imposing alginate/Laponite with 3 and 5 mg/mL graphene oxide to 100% and 1% oscillatory strain during four cycles over 40 min while monitoring storage modulus, with results indicating a complete elastic recovery and insignificant effect of the graphene oxide on the alginate/Laponite bio-ink.

Zeta potential analysis of the prepared hydrogels provides insight when discussing the electrostatic interactions of the nanocomposite hydrogels. According to the literature, the zeta potential of the Laponite is consistently negative in both the acidic and the basic pH range [57]. The zeta potential of the Laponite, which was reported to be around −40 mV, decreases to around −50 mV in the presence of the carboxylate groups of the alginate [28]. Furthermore, adding 3 mg/mL of graphene oxide made the zeta potential even more negative, as illustrated in Figure 2A. In addition, by increasing the concentration of the graphene oxide from 3 to 5 mg/mL, the zeta potential of the nanocomposite hydrogel slightly decreased to the value of −70 mV. This nonsignificant change might be attributed to the existence of negatively charged functional groups, such as carboxyl and hydroxyl anions, in the structure of graphene oxide. The negatively charged functional groups of the graphene oxide have repulsive interactions with the carboxylate groups of the alginate, and the negative charges of the Laponite nanoplatelets shift the zeta potential to a more negative value [58].

#### 2.2.2. Fourier Transform Infrared Spectroscopy

Figure 2B depicts the FTIR spectra of the alginate/Laponite and the nanocomposite hydrogel network with 3 and 5 mg/mL graphene oxide. The incorporation of graphene oxide into the Laponite/alginate structure added specific characteristic peaks to the FTIR spectra of Laponite/alginate at 1040 ${\mathrm{cm}}^{-1}$, which can be attributed to the stretching vibration of the C–O (alkoxy) group in graphene oxide spectra [59]. As shown in Figure 2B, the 1040 ${\mathrm{cm}}^{-1}$ peak became sharper in the hydrogel with a graphene oxide concentration of 5 mg/mL. Similar to the observations in the literature, the characteristic peaks for graphene oxide, representing the stretching vibration of O–H and C=O in COOH, appear at 2349 and 1734 ${\mathrm{cm}}^{-1}$, respectively [60]. Additionally, the characteristic peak of the carboxyl group’s C–O stretching vibration is shifted to the upper wavelengths, so that just one peak is observed at 1468 ${\mathrm{cm}}^{-1}$. This shift may be attributed to the interaction of the Laponite and graphene oxide and has a low intensity in the spectra of the Laponite/alginate hydrogel nanocomposites. Moreover, a band at 1414 ${\mathrm{cm}}^{-1}$ corresponding to asymmetric ${\mathrm{COO}}^{-}$ stretching vibrations of the carboxylate group of structures is observed in all three spectra [61]. Furthermore, asymmetric stretching vibration of the C–O bond of carboxylate groups in the structure of the pure alginate shows itself as a small peak at 1405 ${\mathrm{cm}}^{-1}$, [62] while the C–O stretching mode of the alginate/Laponite hydrogel backbone is observed as two peaks at 1298 and 951 ${\mathrm{cm}}^{-1}$ [63]. Since alginate (0.5 (*w*/*v*)% of gel) is a minor component of the current nanocomposite hydrogel, some of the alginate’s characteristic peaks overlap with the intense peaks of the Laponite. The characteristic peak of the Laponite is observed at 1005 ${\mathrm{cm}}^{-1}$, which is related to the Si–O stretching of its structure [64]. Moreover, the peak observed at 648 ${\mathrm{cm}}^{-1}$ corresponds to the vibration of the Mg–O bond of the Laponite [65]. Similar to the alginate, this peak is also not clear due to the structural electrostatic interaction between the Laponite and graphene oxide. As observed in Figure 2B, all of the samples peaked at 1625 ${\mathrm{cm}}^{-1}$. This peak is due to the bending of the O–H bond found in Laponite, alginate, and graphene oxide. In addition, there is another characteristic peak for the Laponite which appears between 3700 and 3000 ${\mathrm{cm}}^{-1}$, due to the overlap of stretching hydroxyl groups in the Si–OH and Mg–OH bands [66]. This peak, which basically appears at 3600 and 3200 ${\mathrm{cm}}^{-1}$, overlaps with the structural O–H bonds of the alginate.

#### 2.2.3. X-ray Diffraction

The XRD analysis was carried out to prove the presence of graphene oxide nanosheets in the structure of the dried Laponite/alginate hydrogel. As depicted in Figure 2C, the Laponite shows a major peak at 2θ~20.1°, which corresponds to the (100) crystallographic plane with crystal plane spacing of around 14 Å. The Laponite also exhibits some other peaks at 2θ~27.4° and 35.1°, relating to the (005) and (110) crystallographic planes, respectively [67]. Moreover, the XRD pattern of the Laponite/alginate hydrogels, irrespective of graphene oxide incorporation, shows a weak and broad diffraction peak at around 17.32°, indicating a rather amorphous structure for the alginate [68]. The graphene oxide with a layered nanostructure also shows the characteristic small peak at 2θ~12°, relating to the diffraction of the (001) plane, and a spacing obtained from Bragg equations as 10.06 Å. The weak intensity of the diffraction peak of (001) for graphene oxide showed the exfoliation of the graphene layers in the structure of the nanocomposite hydrogel, [69] so that at a lower concentration of 3 mg/mL there is no peak for the (001) plane of the graphene oxide.

#### 2.2.4. Scanning Electron Microscopy

Figure 2D–F demonstrates an SEM image of the fabricated Laponite/alginate bio-ink hydrogel with different concentrations of graphene oxide (0, 3, 5 mg/mL). The pore size of the fabricated hydrogels was analyzed using Image J software. All of the hydrogel-based bio-inks had porous structures, and the porosity of the structures increased from 7.5 ± 2.74 μm to 57.62 ± 12.76 μm with an increase in graphene oxide from 0 to 5 mg/mL. The intrinsic porosity of the Laponite/alginate structure is due to the Laponite, which has a large surface area, forming a dense house-of-cards structure. Additionally, the alginate gives the hydrogel the ability to absorb water, which leads to additional porosities after freeze-drying. Moreover, all the hydrogel samples exhibit a layered microstructure, which indicates the presence of the Laponite as a major phase of the hydrogel. However, as the concentration of the incorporated graphene oxide increases, so does the pore size. This phenomenon may be attributed to the self-organization and the electrostatic interaction between the positively charged Laponite and the negatively surface charged graphene oxide layers [48].

#### 2.2.5. Mechanical Properties of the Bio-Ink

The unique isomorphic substitution of Laponite can enhance the shear-thinning properties, plastic behaviors, and the high elastic response of the hydrogel composites [70]. Specifically, Laponite was shown to significantly improve the shear-thinning properties of alginate and to form a stable colloid with graphene oxide in water [48]. This study presents the rheological optimization of the Laponite/alginate/graphene oxide bio-inks for 3D extrusion-based printing. The mechanical properties of an alginate/Laponite bio-ink with different concentrations of graphene oxide were studied.

The electrostatic interactions between Laponite and graphene oxide lead to a stable colloid [43]. Laponite forms an alkaline environment after dissolving in water, and electrostatic repulsion keeps the Laponite platelets dispersed [53]. Graphene oxide, however, has an acidic pH in water, which could decompose the Laponite hydrogel [57]. The viscosity of Laponite decreases under shear stress and recovers after removing the force, as shown in Figure 2I. The rheological measurement aims to optimize the graphene oxide concentrations to form a mechanically robust yet extrudable bio-ink. Accordingly, Young’s modulus of the bio-inks with different concentrations of graphene oxide, 0, 3, and 5 (mg/mL), respectively, were measured to study the effects of graphene oxide on the mechanical stiffness. The compressive modulus of the bio-inks in the present study was found to be similar to those reported in the literature for alginate/Laponite and graphene oxide/GelMA. For instance, Avery et al. reported that the addition of Laponite to alginate significantly amplified Young’s modulus from 2 to 15 kPa [50]. Based on the literature, Young’s modulus increased from 2 to 7 kPa when reduced-graphene oxide was added to GelMA, and this trend continued until adding 3 mg/mL of the alginate’s reduced-graphene oxide produced a Young’s modulus of 22 kPa. Interestingly, as reported in the literature, increasing the concentration of the reduced-graphene oxide leads to improvements in the mechanical properties of the GelMA hydrogel due to a reduction in the crosslinking density [71]. In addition, the results from these experiments show a 9 kPa increase after adding 5 mg/mL graphene oxide to the alginate/Laponite hydrogel.

Laponite has been reported to improve the shear-thinning properties of alginate [29]. In order to study the shear-thinning properties of the electro-conductive bio-inks, a rheological analysis was performed under various shear stresses (Pa) as a function of the shear rate (1/s). Shear-thinning is a well-known property of Laponite dispersions. This shear thinning is mainly due to the electrostatic repulsion of Laponite’s charged groups forcing the nanoplatelets to form an extended configuration. Simultaneously, the shielding effect of the electrostatic interactions allows the nanoplatelets to fold up, thereby assuming a more compact conformation [72].

As observed in Figure 2G, all the bio-ink formulations show shear-thinning properties and provide a suitable amount of control over the extrusion process. This study showed a negligible difference between the formulation with 3 or 5 mg/mL of graphene oxide. All compositions of the bio-ink showed a sharp increase in shear stress at low shear rates before reaching a plateau at a high shear rate. This trend suggests that manufactured bio-inks inherit the yield stress behavior and rheological properties of Laponite. According to the literature, adding alginate, PEGDA, gelatin, PNiPAM, chitosan, and polyacrylamide does not have a substantial effect on the mechanical properties of the Laponite [73,74].

The shear-thinning properties of the different formulations of the bio-ink can also be determined by measuring the viscosity under constant shear. The viscosity increased with the addition of 3 mg/mL graphene oxide to the alginate/Laponite bio-inks. This increase in viscosity was attributed to the electrostatic interactions between the negatively charged graphene oxide and the positively charged Laponite, as previously mentioned. As shown in Figure 2H, adding 3 mg/mL of the graphene oxide to the alginate/Laponite bio-inks improved the mechanical stiffness and Young’s modulus of the bio-inks. However, the addition of a higher concentration of graphene oxide did not follow a similar trend. Higher concentrations of graphene oxide led to a decrease in the viscosity of the bio-inks under a constant shear rate. As reported in the literature, this trend may be due to the effect of a lower pH on the viscosity of the Laponite [75]. This is primarily because the presence of a higher concentration of ${\mathrm{COO}}^{-}$ at a lower pH leads to changes in the shielding effect of the Laponite platelets, which manifests with less expansion and lower viscosity.

In order to characterize the mechanical integrity of the bio-ink upon printing, the recovery of the nanocomposite hydrogel was measured from high (100% oscillatory strain) to low strain (1% oscillatory strain) conditions. Previously, Laponite and gelatin showed a rapid elastic recovery in less than 10 s [76]. The storage modulus of the bio-ink decreased at 100% strain and illustrated a liquid-like behavior but recovered immediately after the removal of the strain and resumed its solid-like integrity. This rheological test was carried out to compare the effect, in addition to various concentrations, of graphene oxide on the elastic recovery of the bio-inks. Results indicated that the storage modulus was not influenced by increasing the concentration of graphene oxide. Such rapid elastic recovery prevents bio-ink flow after printing and maintains the cylindrical shape of the fibers. These results illustrate the fast elastic recovery of the bio-ink at high and low oscillatory strain after four cycles over 40 min.

### 2.3. Printing and Electrical Conductivity Measurements of the Bio-Ink

As previously mentioned, printing is gaining popularity for fabricating biomimetic scaffolds for tissue engineering and drug screening; however, developing a biocompatible bio-ink remains a challenge [33]. Particular bio-ink requirements vary depending on the final application and the various printing methods, such as extrusion, injection, stereolithography, and laser. The polymer networks of a bio-ink must maintain their mechanical integrity, which is essential for supporting the stability of printed patterns, without compromising their cellular compatibility. Additionally, the viscosity and shear thinning properties of a bio-ink play a crucial role in determining and optimizing the extrusion technique [77,78]. For instance, the viscosity of a shear-thinning bio-ink decreases under shear stress and allows fluent extrusion and prevents cellular damage during printing. Due to their high swelling ratio, hydrogels demonstrate a very low Young’s modulus and mechanical stiffness. Therefore, nanoparticles are incorporated into hydrogels to improve their mechanical properties for producing multifunctional nanocomposite hydrogels [79,80]. Additionally, some tissues such as cardiomyocytes and neurons are electrically conductive, making the addition of electrically conductive material to the bio-ink beneficial [71].

Only a few existing bio-inks are electro-conductive, and even those are only slightly electro-conductive [81]. Metallic and ceramic nanoparticles, such as gold, silver, and graphene oxide, have been added to biocompatible hydrogels to develop electro-conductive bio-inks [33]. Among these nanoparticles, silver and gold have remarkable electro-conductivity. However, they are very expensive and tend to agglomerate and form clusters. Thus, finding cost-effective alternatives with more stable suspension characteristics has gained attention. In this study, we incorporated a graphene oxide mixture to enhance the electrical conductivity of the bio-ink, [82] and Laponite was added to the alginate/graphene oxide to maintain the stability of the graphene oxide colloid in water, and to improve the mechanical properties of the nanocomposite hydrogel [46].

**Figure 3:**
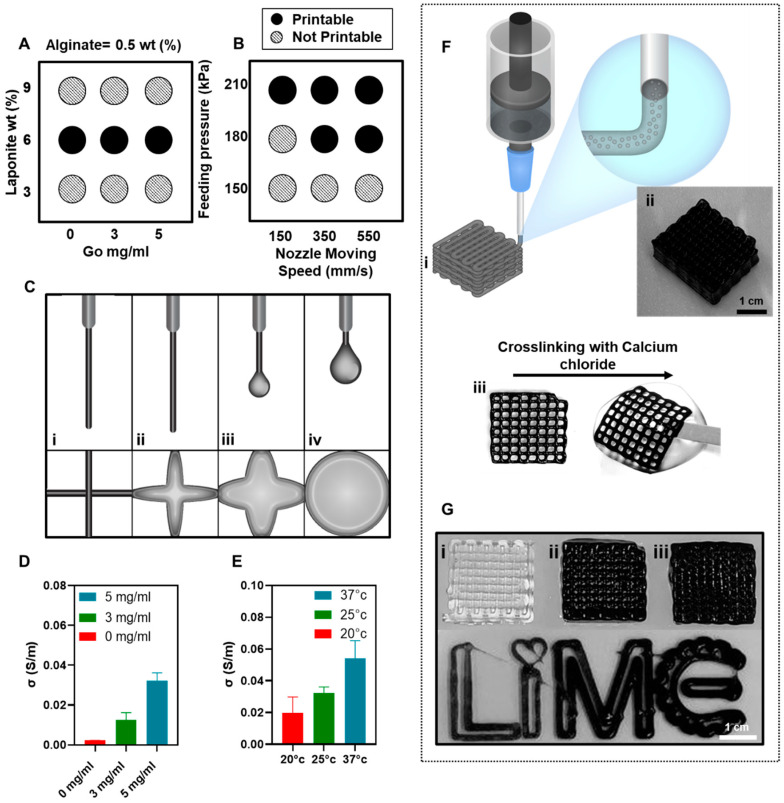
Printability and electrical conductivity of different formulations of the conductive bio-ink. (**A**) Printability of 0.5% (*w*/*v*) alginate, with 3, 6, and 9% (*w*/*v*) Laponite and 0, 3, and 5 mg/mL graphene oxide, respectively, were measured, and 6% (*w*/*v*) Laponite and 5 mg/mL graphene oxide were chosen as an optimum concentration. (**B**) Optimum printing conditions were evaluated and investigated as a function of feeding pressure and nozzle moving speed from 150 to 210 kPa and from 150 to 550 mm/s, respectively, for the optimum formulation of the bio-ink. A feeding pressure of 180 kPa and a nozzle moving speed of 350 mm/s were chosen to print the bio-ink at room temperature. (**C**) Extrusion printing schematic of different formulations of the 0.5%(*w*/*v*) alginate and 3 mg/mL graphene oxide (Go) with 3, 6, and 9% (*w*/*v*) Laponite and the optimized formulation of the bio-ink with 0.5% (*w*/*v*) alginate, 6% (*w*/*v*) Laponite, and 5 mg/mL graphene oxide, respectively, illustrated in order from right to left. This schematic illustrates printing with our bioink and other combinations of polymers and nanocomposites which were not printable. (**D**) The electrical conductivity of alginate/Laponite bio-ink with various concentrations of graphene oxide measured at room temperature. (**E**) Electrical conductivity of the alginate/Laponite with 5 mg/mL graphene oxide measured at 20, 25, and 37 degrees Celsius. (**F**) (**i**) Schematic of extrusion printing, (**ii**) electro-conductive 3D scaffold printed with alginate/Laponite and 5 mg/mL of graphene oxide, (**iii**) rectangular single-layer scaffold crosslinked with 4% ${\mathrm{CaCl}}_{2}$ over 30 min. (**G**) Alginate/Laponite printed with 0 (**i**), 3 (**ii**), and 5 (**iii**) mg/mL graphene oxide, and the color of the 5 mg/mL graphene oxide was observed to be slightly darker than graphene oxide 3 mg/mL.

Figure 3A shows a schematic of the extrusion and fiber formation of the different formulations of the alginate/Laponite bio-ink with various concentrations of graphene oxide. Figure 3A shows the formed filaments of the bio-inks with 0.5% (*w*/*v*), 3% (*w*/*v*) Laponite, and 0, 3, and 5 mg/mL of graphene oxide, respectively. These bio-inks were unsuitable for extrusion printing due to the low mechanical integrity of the stacked layer, or their inability to form consistent and cylindrical filaments following extrusion.

In Figure 3A the bio-ink with 6% (*w*/*v*) Laponite generated cylindrical filaments upon extrusion and provided the desired mechanical integrity for the stacked layer, which hinders the deformation of the pattern. Figure 3A shows the printability chart of the different formulations of the bio-ink. The high viscosity of the alginate/Laponite/graphene oxide bio-inks with 9% (*w*/*v*) Laponite disrupted extrusion, while employing 3% (*w*/*v*) Laponite resulted in droplet formation and the deformation of the stacked-layer after printing.

Thus, the nanocomposite hydrogels with 0.5% (*w*/*v*) alginate, 6% (*w*/*v*) Laponite, and 0, 3, and 5 mg/mL graphene oxide, respectively, were chosen as the optimum formulations of the electro-conductive bio-ink. The feeding pressure and moving nozzle speed are determinative parameters for controlling the printability, filament formation consistency, and fiber diameter. Figure 3B illustrates the printability with the various feeding pressures and nozzle moving speeds. As shown in Figure 3B, a feeding pressure of 180 kPa and a 350 (mm/s) nozzle moving speed were chosen as the optimum conditions for extrusion printing in this work. Figure 3(Cii–Civ) shows the filament formation of the bio-inks with 0.5% (*w*/*v*) alginate, 3% (*w*/*v*) Laponite, and 0, 3, and 5 mg/mL of graphene oxide, respectively. These formulations were unsuitable for extrusion printing due to their low mechanical integrity, the deformation of the stacked-layer, or the formation of a droplet instead of the desired cylindrical filaments following extrusion, as shown in Figure 3(Ci). The electrical conductivity of the alginate/Laponite hydrogel with various concentrations of graphene oxide is presented in Figure 3D. By increasing the concentration of the graphene oxide from 0 to 5 mg/mL, the conductivity of the bio-ink increased to 0.03 (s/m). Additionally, the effect of temperature on the electrical conductivity of the bio-ink with 5 mg/mL graphene oxide is investigated in Figure 3E. The experiment was conducted in the range of 25 °C to 37 °C, which is relevant to the biological applications of these nanocomposite hydrogels as an electro-conductive scaffold. The electrical conductivity of the bio-ink increased with temperature, which is the result of faster electron mobility, ion migration, and, consequently, charge transfer at 37 °C [83]. Figure 3F shows a schematic (i) and optical image (ii) of an extrusion 3D printed cubical pattern with the optimum formulation of the bio-ink. As shown in Figure 3(Fiii), the printed pattern was crosslinked with 4% (*w*/*v*) ${\mathrm{CaCl}}_{2}$. The printed patterns need a crosslinking technique to ensure the maintenance of the complex structure of the material after printing [84]. The crosslinking methods can be different based on the material employed for printing the pattern. The crosslinking reaction for the polymer chains preserves the macromolecular structure from dissolving in water and forms a flexible hydrogel structure, suitable for both tissue engineering and electrical conductor applications. The crosslinking technique for this polymer is an ionic crosslinking with ${\mathrm{CaCl}}_{2}$. The printed samples were immersed in the ${\mathrm{CaCl}}_{2}$ solution for 30 min and then washed with deionized water to remove the excess amounts of ${\mathrm{CaCl}}_{2}$ and to forestall over the crosslinking of the printed pattern. Optical images of the 3D printed cubic patterns from 0.5 (*w*/*v*) alginate, 6 (*w*/*v*) Laponite and 0 (i), 3 (ii), and 5 (iii) mg/mL graphene oxide, respectively, show the printability of different formulations of the bio-ink (Figure 3G).

**Figure 4:**
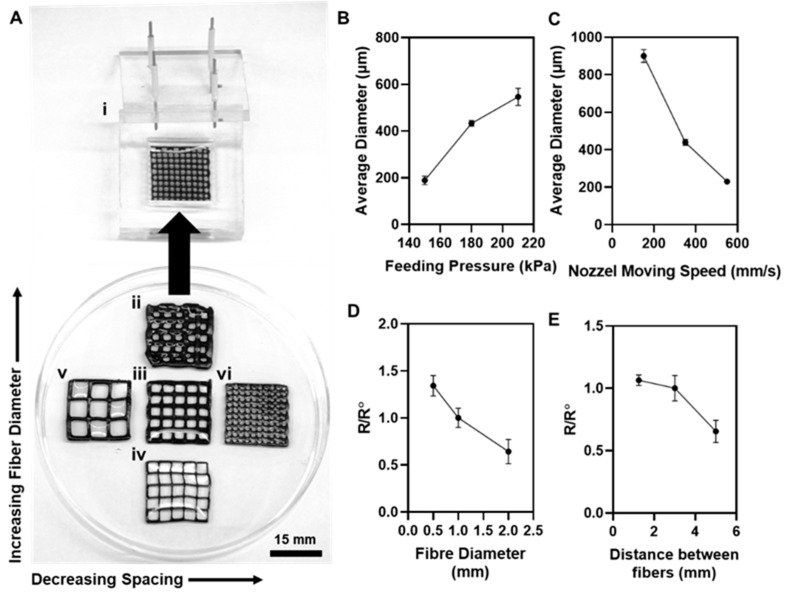
(**A**) Optical images of crosslinked rectangular patterns and (**i**) a polystyrene mold with four copper rods, which was used to measure the electrical resistance of the printed pattern. Optical images of printed rectangular patterns with 2 mm (**ii**), 1 mm (**iii**), and 0.5 mm (**iv**) fiber diameters, with 5 mm (**v**), 3 mm (**iii**), and 1 mm (**vi**) distance between fibers. The average diameter of alginate/Laponite bio-ink with 5 mg/mL graphene oxide was investigated as a function of (**B**) feeding pressure and (**C**) nozzle moving speed. The average diameter of the pattern illustrated an increasing trend by increasing the feeding pressure and reducing the nozzle speed. The electrical conductivity of the printed pattern was measured as a function of (**D**) fiber diameter and (**E**) spacing between fibers. The electrical resistance of the rectangular printed patterns with 0.5 and 2 mm fiber diameters and 1 and 5 mm distance between fibers (R) was monitored and compared with the electrical resistance of a sample with 1 mm fiber diameter and 3 mm distance between fibers ($R_{0}$). By increasing fiber diameter and distance between fibers, electrical conductivity of the printed patterns decreased.

A polystyrene mold was designed and fabricated to maintain the consistency of measuring the electrical resistance of the printed patterns. Four copper rods were fixed inside the nonconductive lid and were connected to a multimeter. The patterns were crosslinked and placed inside the mold to measure the resistance of the samples. Figure 4A shows a rectangular printed pattern inside the mold. Rectangular patterns were printed with various fiber diameters, including, 0.5, 1, and 2 mm with different spacing between fibers, including, 1, 3, and 5 mm, so to compare the effect of geometry on the electrical resistance of the printed patterns. Figure 4A shows a rectangular pattern with 2 mm (ii), 1 mm (iii), and 0.5 mm fiber diameters (iv), and 3 mm spacing between fibers. The 1 mm, 2 mm, and 3 mm spacing between fibers, and the 1 mm fiber diameters are shown in Figure 4 (Av,Aiii,Avi), respectively.

Various fiber diameters were printed in order to show the adaptability of the bio-ink for printing and to demonstrate the possibility of the extrusion printing of narrow lines. This was accomplished by adjusting the feeding pressure. As shown in Figure 4B, by decreasing the feeding pressure from 210 to 150 kPa, the diameter of the fibers was reduced to an average of 200 µm. However, decreasing the nozzle moving speed results in an increase of the fiber’s diameter to 900 µm, as shown in Figure 4C. A wide range of fiber diameters was achieved by changing the printing parameter, which shows the robust control and the adaptability of the printing process with this bio-ink. The electrical resistance of the various patterns was measured (R) and compared with the initial pattern, which was chosen to be a rectangular pattern with a 1 mm fiber diameter and a 3 mm fiber distancing ($R_{0}$). The electrical resistance of the different fiber diameters and the spacing between the fibers were measured and compared with the electrical resistance of the initial sample. As shown in Figure 4D, increasing the fiber diameter from 0.5 to 2 mm results in a decrease in the electrical resistance and an increase in the conductivity of the pattern, which is due to the existence of a wider channel for transferring the ions and electrons in a rectangular pattern. On the other hand, reducing the fiber spacing leads to an increase in the electrical resistance and a decrease in the electrical conductivity of the pattern. The ions are responsible for transferring the electrical current and introducing more branches to the rectangular pattern results in spreading the ions into more channels and weakening the current in each filament. Consequently, the adaptability of the bio-ink for printing provides more control over the electrical characteristics.

### 2.4. Design and Fabrication of the Flexible Conductor on Alginate Substrate

In this work, a new technique was introduced for ensuring the adhesion of the printed conductor to the surface of the alginate sheet, and the alginate was employed to fabricate a flexible, biodegradable, and biocompatible substrate. Alginate is an anionic polymer that is naturally extracted from brown seaweed [23]. Alginate has found various applications in wound healing, controlled release, targeted drug delivery, and tissue engineering due to its biocompatibility, swelling capacity, sol–gel transition, mechanical stability, and viscoelastic properties [85].

Due to its favorable properties, alginate was chosen to produce a flexible substrate. The fabrication of the alginate substrate improves the mechanical stability of the conductor and extends the bio-ink’s use in targeted drug delivery and wound healing applications. The fabrication ease and repeatability of the process contrasts with the challenges associated with other techniques. Extrusion printing is the strategy we employed to print a 3D complex conductor on the alginate substrate. An electro-conductive bio-ink has been employed to print patterns on the alginate substrate. This conductive bio-ink consists of Laponite, alginate, and graphene oxide so to maintain the mechanical integrity of the bio-ink after printing, improve the viscoelasticity, and create electrical conductivity. This nanocomposite hydrogel is biocompatible and has the requisite swelling capacity, a good electrical conductivity, and the viscoelastic properties necessary for practical applications.

**Figure 5:**
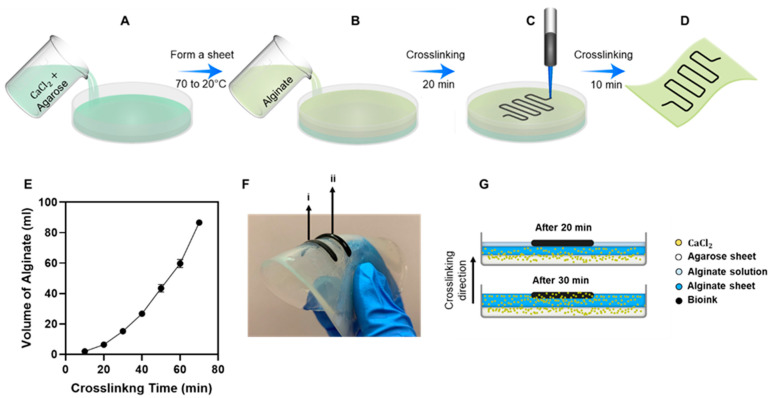
Schematic of the fabrication process of printing a conductive pattern on the alginate sheets. (**A**) Preparation of agarose sheet with 4% (*w*/*v*) ${\mathrm{CaCl}}_{2}$. (**B**) Spreading the alginate solution at the top of the agarose sheet for the slow diffusion of ${\mathrm{CaCl}}_{2}$ from the agarose sheet into the alginate for 20 min. (**C**) Printing the electrical-conductive pattern on the alginate sheet, which was formed inside the alginate solution during the 20 min of crosslinking. (**D**) Parallel crosslinking of the alginate solution and bio-ink for 10 min to form the final substrate. (**E**) Crosslinking profile of various volumes of alginate solution was measured to find the optimum time for the crosslinking of the alginate substrate in a 100 mm Petri dish. (**F**) Optical image of printed bio-ink on the alginate substrate after 20 min (i) and 30 min (ii) of crosslinking. (**G**) Schematic of the crosslinking process of the alginate substrate and the bio-ink from bottom to the top illustrates the migration of ${\mathrm{Ca}}^{2+}$ ions from the agarose sheet to different levels of alginate over time.

In order to fabricate the alginate substrate, 1.5% (*w*/*v*) agarose solution was added to 4% (*w*/*v*) of ${\mathrm{CaCl}}_{2}$ and heated to 70 °C to dissolve the agarose. The solution was subsequently cooled to room temperature to form a sheet in a 100 mm Petri dish, as shown in Figure 5A. Then, 4% (*w*/*v*) alginate solution was added to the top of the agarose sheet, as shown in Figure 5B, and crosslinked for 20 min. Bio-ink was printed on the alginate solution and on top of the crosslinked alginate layer so to support and fix the printed pattern, as shown in Figure 5C. The crosslinking continued for 10 min to fully ensure the attachment of the printed pattern to the alginate sheet by forming covalent bonds between calcium ions and alginate [86], as shown in Figure 5D. The optimum crosslinking time of 15 mL, and alginate 4% (*w*/*v*) with 4% (*w*/*v*) of ${\mathrm{CaCl}}_{2}$, was calculated using Figure 5E. As observed in Figure 5F, the bio-ink was printed on the alginate solution after 20 (i) and 30 (ii) minutes of crosslinking of the alginate sheet. The samples (ii) did not show sufficient attachments between the bio-ink and the alginate sheet, and the pattern piled off after the first round of stretching. As shown in Figure 5G, the flexible fiber was printed on the solid surface of the half-crosslinked alginate solution. The fiber was trapped inside the alginate solution and crosslinked in parallel with the solution. This method provided a strong attachment between the alginate sheet and the flexible fiber. This flexible pattern remained stable on the alginate sheet even after 10 rounds of stretching.

**Figure 6:**
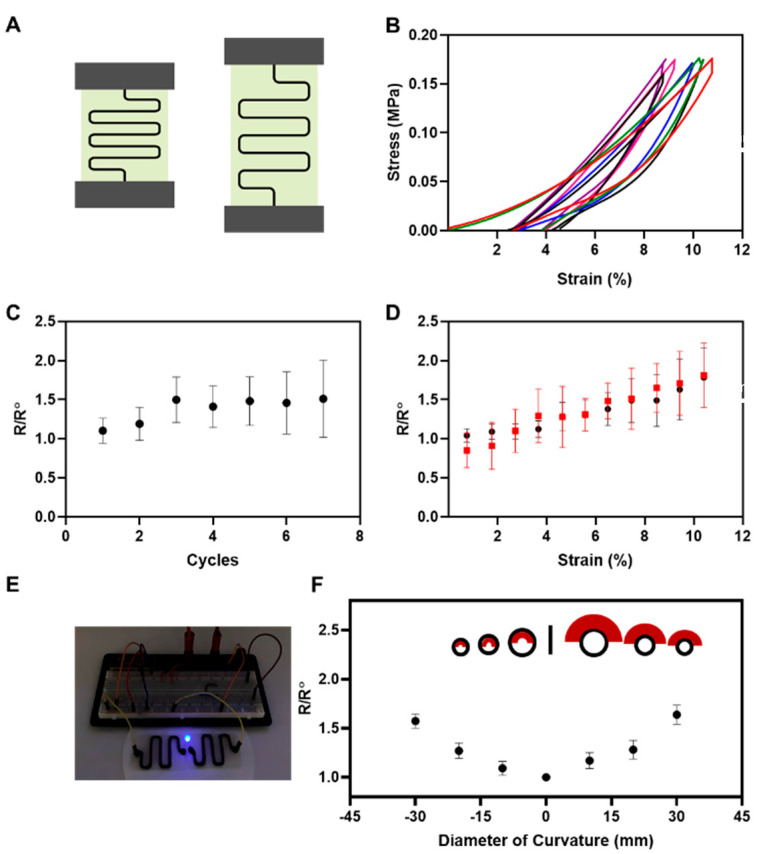
Elasticity, flexibility, and electrical resistance of the printed pattern on alginate sheets. (**A**) Schematic of the setup used for stretching the sample, measuring electrical resistance, and monitoring stress as a function of strain to measure the tensile properties and electrical conductivity of the sample at different cycles. (**B**) The sample was stretched by a maximum of 10% of its original length and recovered with a rate of one cycle/minute and then released to its original length, and this process repeated over seven cycles to report more than 70% stress recovery after each stretch. (**C**) Durability of the printed pattern over 7 cycles. The effect of the cyclic load was monitored by measuring the electrical resistance of seven cycles after each stretch (R) compared to the electrical resistance of the upstretched sample ($R_{0}$), and the cyclic resistance measurement showed an insignificant increase in the electrical resistivity. (**D**) Durability of the printed pattern over 1 cycle with various strains. The sample was stretched by 10% of the original length in a cycle, and the electrical resistance was measured by exposing the sample to 1–10% strain to monitor a complete recovery of the sample after removing the load. (**E**) The optical image of conductivity properties of the flexible conductor was tested by a 3 V LED bulb experiment. (**F**) Schematic and diagram of a printed pattern of the bio-ink on alginate sheets. The samples were wrapped around a cylindrical mold with various curvature diameters to measure the electrical resistance of the printed bio-ink after cyclic load (R) to initial electrical resistance ($R_{0}$ ), reducing the curvature diameter results in increasing the resistivity, and, consequently, decreasing the electrical conductivity of the printed samples.

The functionality of an elastic conductor relies on its ability to maintain its electrical conductivity under various loading conditions such as stretching, as shown in Figure 6A, and bending, as shown in Figure 6E. The elastic printed conductor on the alginate sheet illustrated outstanding mechanical stretchability without any observable cracking or detachments from the thin alginate substrate, as observed in Figure 6B. As illustrated in Figure 6C, the negligible increases in the electrical resistance of the printed pattern after each cycle of force application and removal indicate the exceptional electrical conductivity recovery of the material. The electrical resistance of the flexible conductor was monitored over the first one-minute cycle of applying 10% strain. The results indicate a steady increase and recovery of the electrical resistance by reducing the strain to 0%. The results from Figure 6D indicate the significant elastic recovery and sustainable electrical conductivity of the printed conductor and illustrate the strong adhesion of the conductor to the alginate substrate. The unique structural features of the flexible conductor result in developing durable elastic conductors for biomedical application. To further characterize the physical properties and realize the excellent performance of the flexible conductor, the electrical resistance was observed at different loading conditions.

As shown in the schematic, Figure 6A, sheets of alginate were glued to two pieces of garnet sandpaper from two ends, and the sandpapers were fixed inside two grips of the tensile machine. This platform eliminated any direct contact between the alginate sheets and grips so to forestall the unwanted breakage of the substrate. A 10 kN% load was applied to each sample to stretch them by 10% of their original length. This experiment was repeated for seven cycles with a rate of one cycle/min to investigate the elastic recovery of the conductor, as seen in Figure 6B. The stress–strain curves indicated slight variations after the second cycle; however, the elastic recovery remained intact after seven cycles. We also measured the electrical resistance after each cycle to illustrate the durability of the conductor.

The result from Figure 6C indicated no significant differences in the electrical resistance of a sample after each cycle. Figure 6D reveals the variations in the electrical resistance during and after each cycle, which were measured to illustrate the full recovery of the electrical conductivity of each sample at the end of the cycle. After removing the force from each sample, no significant deformation was observed, and the electrical resistance of the stretched sample recovered to the resistance of the unstretched sample. The elasticity and durability of the conductor were confirmed by only slight changes in the electrical resistance within the seven cycles. In addition, the conductivity of the flexible conductor is shown in Figure 6E. We also studied the repeatability of the fabrication process by investigating the electrical resistance of the triplicated samples. In order to investigate the durability of the conductor, alginate sheet samples were bent to a maximum curvature diameter of 30 mm. These results represent the ability of this flexible conductor to provide conformal contact with skin and accommodate natural motions without significant restraint in mechanical properties and electro-conductivity [87]. Figure 6F illustrates the flexibility and durability of the conductor on the alginate sheet. The samples were wrapped around a curved shape and the electrical resistance of the sample was investigated at that position. Data from Figure 6F show a slight increase of the electrical resistance after bending each sample with various diameters, including 10, 20, and 30 mm. Although the results showed a slight increase of the electrical resistance by increasing the curvature diameter, bending did not have a noticeable effect on the electrical conductivity of the samples.

**Figure 7:**
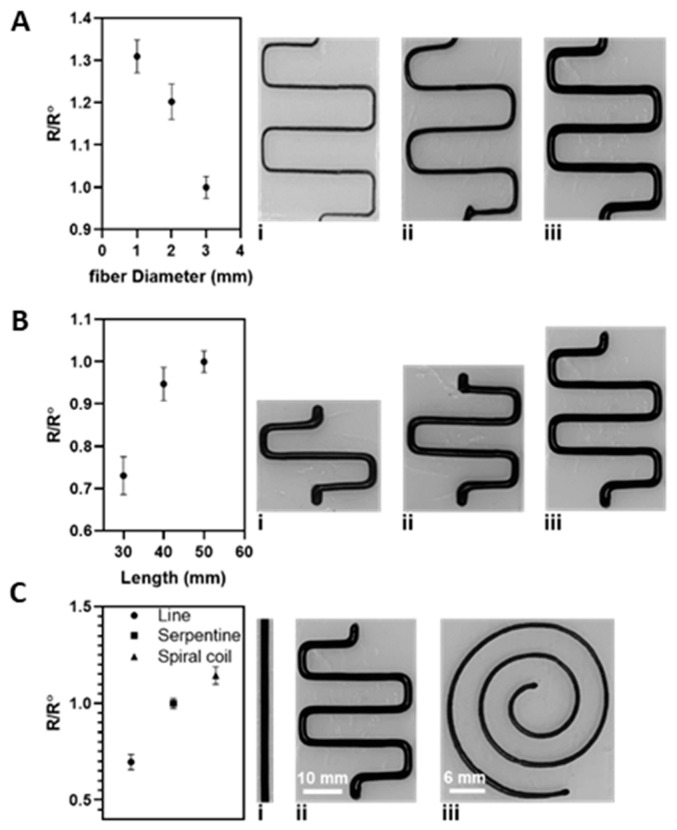
The electrical resistance of the serpentine pattern was investigated as a function of various (**A**) fiber diameters ((**i**)-1mm, (**ii**)-2mm, (**iii**)-3mm)and (**B**) fiber lengths, while variations of the resistance indicate an increasing trend by decreasing the fiber diameter and show an increase by shortening the serpentine pattern ((**i**)-30mm, (**ii**)-40mm, (**iii**)-50mm). (**C**) Switching the printed pattern to a straight line and a spiral design introduces a change in the resistivity of the samples and reveals the repeatability of the fabrication process ((**i**)-line, (**ii**)-serpentine, (**iii**)-spiral).

Variations in electrical resistance were measured for various fiber diameters, lengths, and patterns. The electrical resistance of a serpentine pattern with 50 mm length and 3 mm fiber diameter was measured as an initial value ($R_{0}$), and the electrical resistance of the samples with 30 and 40 mm length and 1 and 2 mm fiber diameter were measured as (R) and compared with the initial value. Figure 7A shows that due to the wide channel for transferring the ions from one end of the pattern to the other, the electrical resistance was reduced when the fiber diameter increased from 1 to 3 mm. Similarly, the electrical resistance of the printed pattern decreased when the length of the pattern was also decreased, as shown in Figure 7B. The repeatability of the fabrication process was also investigated by printing various printed patterns, including a straight line, a serpentine, and a spiral pattern, as shown in Figure 7C.

## 3. Conclusions

We have successfully fabricated a novel electro-conductive bio-ink for developing soft 3D printed hydrogels with various potential applications, from tissue engineering to soft-based bioelectronics. The novel bio-ink is composed of alginate as the base material loaded with Laponite nanoplatelets and reduced-graphene oxide. The resulting bio-ink exhibited superior electrical conductivity and viscoelastic properties as compared to pristine alginate. The shear-thinning properties of the bio-ink were evaluated using rheological tests, while the stretchability and durability of the 3D printed meshes with different fiber diameters were examined under significant stretching and bending at room temperature. Our results suggest that the addition of graphene oxide to alginate/Laponite bio-ink did not compromise the shear-thinning properties of the bio-ink. As such, the bio-ink was successfully used for creating complex conductive patterns on a hydrogel substrate. The conductive patterns showed consistent electro-conductive properties under seven cycles of stretching, and the reproducibility of the manufacturing process was examined by printing various patterns with different fiber diameters. Overall, the proposed novel bio-ink holds promise for designing implantable microelectronics with mechanical properties that match those of the host tissue and are suitable for accommodating body movement.

## 4. Materials and Methods

Materials: Alginic acid sodium salt (alginate), graphene oxide, gelatin from porcine skin (type A, 300 bloom gel strength), agarose, and calcium chloride were all obtained from Sigma-Aldrich, St. Louis, Missouri. Laponite XLG, a synthetic nanosilicate, was acquired from BYK Additives, Germany.

Preparation of Bio-inks: A total of 0.5% (*w*/*v*) alginate, 6% (*w*/*v*) Laponite, and 5 mg/mL graphene oxide were prepared by, first, dispersing the graphene oxide in deionized water. The suspension was then sonicated for 5 min, alginate was dissolved in the graphene oxide suspension, and the solution was cooled to 5 ℃. Laponite was added to the solution and immediately vortexed for 5 min to ensure the homogenous distribution of Laponite nanoplatelet in the solution. In order to prepare different formulations of the bio-ink, different concentrations of the graphene oxide and Laponite were added to the solution, and the rest of the procedure was followed as explained above. A list of the different formulations of the bio-ink is given in Table 1.

Zeta Potential: Zeta potential tests were conducted using a Brookhaven BI-ZR3 Zeta Potential Analyzer with a 600 nm wavelength laser at room temperature. Samples with 0.5% (*w*/*v*) alginate, 6% (*w*/*v*) Laponite, and 0, 3, 6% (*w*/*v*) graphene oxide, respectively, were diluted to 0.2% (*w*/*v*) and sonicated for 5 min to ensure a uniform distribution of graphene oxide and Laponite. This test was conducted to study the effect of the introduction of different concentrations of graphene oxide on the zeta potential of the bio-ink.

Fourier Transform Infrared Spectroscopy (FTIR): The chemical structure of Laponite/alginate with various concentrations of graphene oxide was analyzed by a PerkinElmer Spectrum Two Fourier transform infrared spectrometer (USA) to obtain FTIR spectra by a potassium bromide (KBr) technique. Samples were firstly freeze-dried and then ground into powder and mixed with KBr to collect the spectra and determine the interactions between the components within the range of 400–4000 ${\mathrm{cm}}^{-1}$.

X-ray Diffraction (XRD): Firstly, samples were freeze-dried and ground into a powder. The crystal structure of the bio-ink with different concentrations of graphene oxide was analyzed using a PANalytical Empyrean X-ray Diffractometer at room temperature with a current of 40 mA and a voltage of 45 kV over the diffraction angle range of 10°–90° (2θ).

Scanning Electron Microscopy (SEM): SEM images were acquired using the Hitachi S-4800 FESEM microscope at an accelerating voltage of 1 kV. Samples were freeze-dried by storing them in a −80 °C freezer for 24 hr, lyophilized for 2 days, tapped to the SEM stubs, and then coated with gold–palladium. SEM images were employed to investigate the microstructural differences between bio-ink formulations with various concentrations of graphene oxide (0, 3, and 5 mg/mL), with 0.5% (*w*/*v*) alginate and 6% (*w*/*v*) Laponite.

Rheometer and Mechanical Analysis: The Young’s modulus, storage modulus, shear stress, and viscosity of the bulk bio-inks with different concentrations of the graphene oxide (0, 3, 5 mg/mL) were measured using an Anton Paar MCR 502 rheometer (Graz, Austria) over shear rate sweeps of 0.001 to 10 Hz at room temperature. Viscosity was measured under a constant shear rate over 300 s, and stress recovery measurements were conducted at 1 Hz by alternating the application of 100% strain and 1% strain for 5-min intervals on the bulk bio-inks.

3D printing Process: All 3D printing experiments for the present work were conducted employing a Cellink Inkredible+ (Cellink, Sweden) microextrusion 3D printer with pneumatic pressure for extruding materials. Repetier and Slic3r software were used to manually generate codes for the 3D printing of rectangular patterns. In order to print serpentine and spiral patterns, AutoCAD was employed to develop the drawings and Cura was used to develop the final codes for 3D printing. Different formulations were used to study the printability of the bio-inks (Figure 2B). In order to provide the requisite control over the diameter of printed fibers, the print head nozzle moving speed was optimized with different printing air pressures for feeding pressure (Figure 2C). The nozzle moving speed and the printing feeding pressure were determined for the bio-ink with 6% (*w*/*v*) Laponite, 5 mg/mL graphene oxide, and 0.5% (*w*/*v*) alginate. The electro-conductive bio-inks with alginate 0.5% (*w*/*v*), Laponite 6% (*w*/*v*), graphene oxide 5 mg/mL, and 2% (*w*/*v*) ${\mathrm{CaCl}}_{2}$ were used to print the patterns for this study. Three different feeding pressures: 150, 180, 210 (kPa) and nozzle moving speeds: 150, 350, 550 (mm/s) were employed to optimize the printing process. After optimizing the printing process, the Repetier software was used to customize the codes, and the patterns were printed with three different spacings between the printed fibers, including 1, 3, and 5 mm (see Figure 2G). Besides, three different needle gauges of 18, 22, and 25 with an inner diameter of 0.84, 0.41, and 0.26 mm, respectively, were used to produce fiber sizes of 0.5, 1, and 2 mm for the rectangular patterns. All the patterns were allowed to crosslink with 2% ${\mathrm{CaCl}}_{2}$ for 30 min at room temperature. The electrical conductivity of the rectangular pattern was measured using a multimeter at room temperature.

3D Bioprinting on Alginate Substrate: A 4% (*w*/*v*) alginate was chosen to develop a flexible substrate for printing an electrically conductive pattern. The first step to develop a flexible printed pattern was spreading the 4% (*w*/*v*) alginate solution in a 100 mm Petri dish and crosslinking it with 4% (*w*/*v*) ${\mathrm{CaCl}}_{2}$. New technology was developed in order to provide a sufficient attachment between the electrical-conductive printed pattern and the alginate sheet. The following method was employed to fabricate the flexible substrate. A 1.5% (*w*/*v*) agarose solution was prepared by suspending 1.5 g agarose powder in 100 mL deionized water and dissolving at 70 °C to form a clear solution. A 4% (*w*/*v*) ${\mathrm{CaCl}}_{2}$ solution was then added to the clear agarose solution, and 15 mL of the final solution was spread in a 100 mm Petri dish and cooled down to room temperature to form a flat sheet of agarose containing ${\mathrm{CaCl}}_{2}$. Then, 15 mL of 4% (*w*/*v*) alginate was spread to the same Petri dish at the top of the agarose sheet. The agarose sheet containing 4% (*w*/*v*) ${\mathrm{CaCl}}_{2}$ was employed to provide more control over the crosslinking process of the alginate sheet by eliminating the nonuniform topography of the alginate sheets as a result of direct contact with ${\mathrm{CaCl}}_{2}$ solution and adjusting the crosslinking time by changing the concentration of the ${\mathrm{CaCl}}_{2}$ solution.

After preparing the alginate sheet, developing the printing codes, and choosing an optimum formulation of the bio-ink, different patterns were printed on the substrate. The most significant challenge in this process was trapping the printed pattern inside a half-crosslinked solution to provide a strong attachment between the printed patterns to the alginate sheet. Therefore, the prolonged crosslinking method played a significant role in providing control over the crosslinking procedure. The various volumes of alginate solution were prepared and crosslinked with a 1.5% (*w*/*v*) agarose sheet containing 4% (*w*/*v*) ${\mathrm{CaCl}}_{2}$, and used as the solution to monitor the crosslinking velocity (mm/min). After exposing the 4% (*w*/*v*) alginate solution to the agarose sheet for 20 min, 2 mm of the alginate solution was crosslinked to provide sufficient support and to maintain the printed pattern on the alginate sheet. Partially crosslinking the alginate solution is necessary for forming layers of alginate sheets from the bottom to the top of the solution. The layers of alginate sheets tolerate the weight of the bio-ink and prevent any deformations of the bio-ink after printing on alginate and eliminate sinking the pattern to unwanted levels of alginate substrate. After printing the pattern, the sample was crosslinked for 20 more minutes so to be fully crosslinked. During the next 20 min, the printed patterns and the alginate sheet were crosslinked in parallel.

Electrical Conductivity Measurement: The electrical conductivity of the rectangular patterns was measured using four conductive copper rods with a 1 mm diameter. The distance between every two sets of rods plays an important role in keeping the consistency of the measurement. AutoCAD was used to develop a drawing, and the sample was later laser cut to form the mold.

Besides, a spiral pattern was chosen to monitor the variations of electrical resistance of different fiber diameters, lengths, and printing patterns on the alginate sheets. In addition, electrically conductive bio-ink was printed in three different patterns on the alginate sheets to study the effect of the printing patterns on the electrical resistance of the samples.

Tensile Test: Mechanical properties of the serpentine pattern on alginate sheets were measured using MTI Electromechanical Universal Testing Machine Systems with 1 kN load cell. In order to secure the alginate sheet inside the grips, the samples were cut into rectangular shapes (10 cm × 5 cm) length and glued to sandpaper from two ends, and then the sandpapers were fixed inside two grips. This method successfully prevents any undesired ruptures in the prepared samples as a result of direct contact with the alginate sheets and the grips during the measurement. For the cyclic tensile test, the samples were stretched with a linear velocity of 1 mm/min and the test was repeated for 7 cycles with a maximum strain of 10%. The electrical conductivity of the printed pattern on the alginate sheets was measured after each cycle and during the first cycle using a multimeter.

Cell culture: U251 Glioblastoma cell lines were cultured in T-25 flasks in a 5% CO_2_ incubator at 37 °C. Dulbecco’s modified Eagle’s medium (DMEM; Gibco, Sigma-Aldrich, St. Louis, MO, USA) supplemented with 10% FBS (Invitrogen 16000), 100 IU/mL of penicillin, and 100 mg/mL of streptomycin was used as the cell media and was changed every other day. Cells were cultured up to 90% confluency. Afterwards, the U251 cells were detached into a single cell suspension followed by centrifuging and diluting, and ready for counting using a hemocytometer to obtain a certain number of cells.

Cell viability assay: To measure the cell viability of the fabricated hydrogels with different GO concentrations, 96-well plates (VWR, Radnor, PA) were seeded with 10,000 cells per well and cultured in 0.2 mL of media for 24 h. Presto blue (PB) assay was used as a standard method for rapidly evaluating the viability and proliferation of a wide range of cell types by measuring the metabolic activity of the live cells. An indirect extraction method was used to assess the cell viability of the experimental samples. Equal weights (0.3 g) of GO-incorporated hydrogels in triplicate were incubated in 2 mL DMEM with 10% *v*/*v* of fetal bovine serum at 37 °C, in 5% CO_2_ and fully humidified air for 1, 3, 7 days, respectively. The liquid extraction medium (500 µL each) was collected for the PB assay at each time interval and was substituted with the fresh DMEM + FBS solution. Then, 200 µL of each periodically collected liquid extract was added to each well and incubated for 24 h. Finally, the cell metabolic activity was determined using a PB cell viability reagent (Invitrogen, A13261, Waltham, MA, USA). PB reagent was added to each well at 10% concentration of the total well volume, and the plate was incubated for 15 min. Excitation and emission wavelengths of 560 and 590 nm were used to measure the fluorescent intensity of the incubated cells. Subsequently, the viability of the samples was calculated using the following equation:

Cell Viability: (Average fluorescent density of the samples/Average fluorescent density of the control) ∗ 100.

## Figures and Tables

**Table 1:** A list of different formulations of the bio-ink.

Sample Code	Laponite (wt)%	Graphene Oxide (wt)%
**S1**	3	0
**S2**	3	3
**S3**	3	5
**S4**	6	0
**S5**	6	3
**S6**	6	5
**S7**	9	0
**S8**	9	3
**S9**	9	5

All the formulations have 0.5 wt% Alginate.

## Supplementary Materials

The following are available online at https://www.mdpi.com/article/10.3390/gels7040240/s1, Figure S1: Presto blue cell viability assay of the nanocomposite hydrogels with different graphene oxide (GO) concentrations.
