# Supplementary material for: Silicate-Based Electro-Conductive Inks for Printing Soft Electronics and Tissue Engineering
Source: Gels. 2021 Nov 27;7(4):240. doi: 10.3390/gels7040240 (PMC8702023; doi:10.3390/gels7040240)
Supplement: Supplementary file 1 [file gels-07-00240-s001.zip › gels-1460491-supplementary.pdf]

Supplementary Information

Silicate-Based Electro-Conductive Inks for Printing Soft Electronics and Tissue Engineering

Sadaf Samimi Gharaie [1], Amir Seyfoori [1,2], Bardia Khun Jush [1], Xiong Zhou [1], Erik Pagan [1], Brent Godau [1] and Mohsen Akbari [1,2,3,4,*]

[1] Laboratory for innovations in Microengineering (LiME), University of Victoria, Victoria, BC, V8P 5C2, Canada; s.f.sadaf@gmail.com (S.S.G.); am.seyfoori@gmail.com (A.S.); bkhunjus@uvic.ca (B.K.J.); sina81@gmail.com (X.Z); erikpm@uvic.ca (E.P.); brentgodau@gmail.com (B.G.).

[2] Center for Advanced Materials and Related Technologies (CAMTEC), University of Victoria, Victoria, BC, V8P 5C2, Canada

[3] Biotechnology Center, Silesian University of Technology, 2A, 44-100 Gliwice, Poland

[4] School of Biomedical Engineering, University of British Columbia, Vancouver, BC, V6T 1Z4, Canada

* Correspondence: makbari@uvic.ca

Figure S1

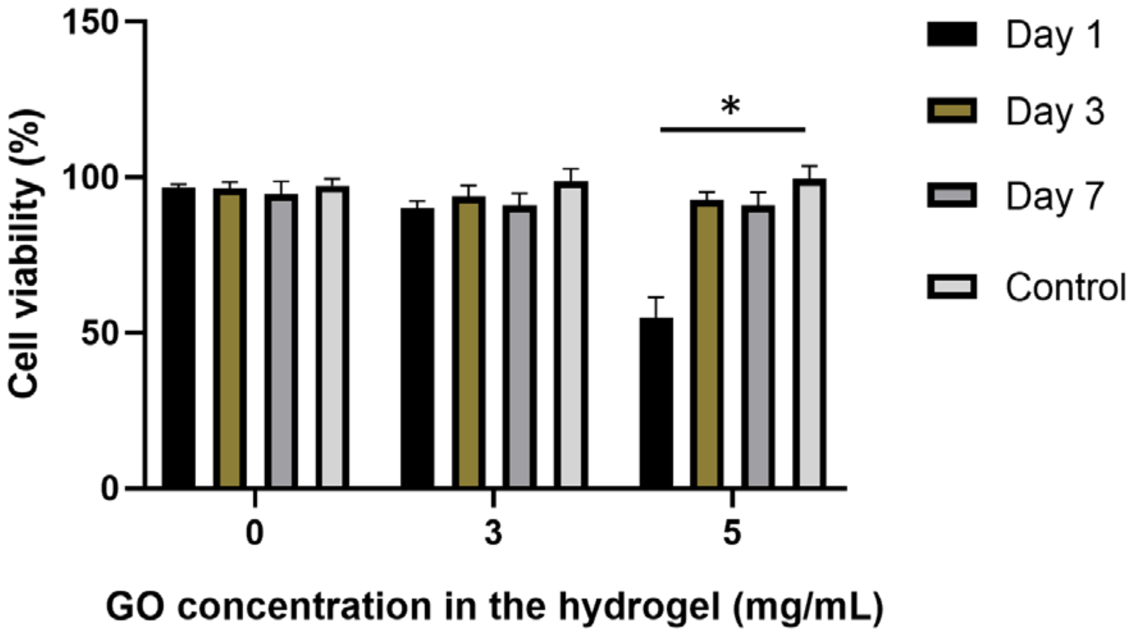

Figure S1. Presto blue cell viability assay of the nanocomposite hydrogels with different graphene oxide (GO) concentrations. ($n = 3$), * $p < 0.05$.
